# Feeding rates of *Balloniscus sellowii* (Crustacea, Isopoda, Oniscidea): the effect of leaf litter decomposition and its relation to the phenolic and flavonoid content

**DOI:** 10.3897/zookeys.176.1940

**Published:** 2012-03-20

**Authors:** Camila Timm Wood, Carolina Casco Duarte Schlindwein, Geraldo Luiz Gonçalves Soares, Paula Beatriz Araujo

**Affiliations:** 1 Universidade Federal do Rio Grande do Sul, Departamento de Zoologia, Laboratório de Carcinologia, Av. Bento Gonçalves, 9500, pr. 43435, 91501-970, Porto Alegre, RS, Brazil; 2 Universidade Federal do Rio Grande do Sul, Departamento de Ecologia, PPG Ecologia, Av. Bento Gonçalves, 9500, pr. 43422, 91501-970, Porto Alegre, RS, Brazil; 3 Universidade Federal do Rio Grande do Sul, Departamento de Botânica, Laboratório de Ecologia Química e Quimiotaxonomia, Av. Bento Gonçalves, 9500, pr. 43423, 91501-970, Porto Alegre, RS, Brazil

**Keywords:** Woodlice, digestibility, total phenolics, flavonoid concentration, consumption rate, assimilation rate

## Abstract

The goal of this study was to compare the feeding rates of *Balloniscus sellowii* on leaves of different decomposition stages according to their phenolic and flavonoid content. Leaves from the visually most abundant plants were offered to isopods collected from the same source site. *Schinus terebinthifolius*,the plant species consumed at the highest rate, was used to verify feeding rates at different decomposition stages. Green leaves were left to decompose for one, two, or three months, and then were offered to isopods. The total phenolic and flavonoid contents were determined for all decomposition stages. Consumption and egestion rates increased throughout decomposition, were highest for two-month-old leaves, and decreased again in the third month. The assimilation rate was highest for green leaves. The mode time of passage through the gut was two hours for all treatments. Ingestion of leaves occurred after two or three days for green leaves, and on the same day for one-, two- and three-month-old leaves. The speed of passage of leaves with different decomposition stages through the gut does not differ significantly when animals are fed continuously. However, it is possible that the amount retained in the gut during starvation differs depending on food quality. The digestibility value was corrected using a second food source to empty the gut of previously ingested food, so that all of the food from the experiment was egested. The digestibility value was highest for green leaves, whereas it was approximately 20% for all other stages. This was expected given that digestibility declines during decomposition as the metabolite content of the leaves decreases. The phenolic content was highest in the green leaves and lowest in three-month-old leaves. The flavonoid content was highest in green leaves and lowest after two months of decomposition. Animals ingested more phenolics when consumption was highest. The estimated amount of ingested flavonoids followed the same trend as assimilation rate. Flavonoids accounted for a large portion of total phenolics, and the estimated amount of flavonoids consumed was similar for one-, two- and three-month-old leaves. Our results suggest that the high phenolic and flavonoid concentrations in green leaves are feeding deterrents. Isopods may discriminate among concentrations of flavonoids and modify their consumption rates to maintain their intake of flavonoids when ingesting leaves with lower flavonoid content.

## Introduction

Litter dynamics are of great importance in ecosystem functioning and are influenced by many different organisms. Isopods, earthworms, lumbricids, diplopods, dipteran larvae, and termites are detritivores of organic soil litter that play a major role in the cycling of nutrients, which is an important ecosystem service. Detritivores have low assimilation efficiency (Szláveczs and Pobozny 1995), and thus contribute to leaf litter decomposition indirectly by returning large amounts of consumed litter as feces (Quadros and Araujo 2008), which provides increased surfaces that are readily colonized by microbial populations (Hassall et al. 1987, Loureiro et al. 2006).

Detritivores exhibit feeding preferences that may be related to leaf senescence (Wieser 1984, Hassall et al. 1987, Yeates and Barmuta 1999), the nutrient content of food (Graça et al. 2001), microbial colonization (Gunnarsson 1987, Kautz et al. 2000, Zimmer et al. 2003, Ihnen and Zimmer 2008), and the presence of unpalatable or indigestible compounds (Cameron and LaPoint 1978, Hassall and Rushton 1984, Target et al. 1986, Souza et al. 1998, Canhoto and Graça 1999, Lambdon and Hassall 2005). The contribution of isopods to decomposition depends on leaf litter degradation and may be influenced by food preference (Van Wensem et al. 1993).

Frequent switching between different types of food and regulating the intake of specific defensive chemicals are behavioral mechanisms used to accommodate for chemically defended foods (Glendinning 2007). Whenever possible, isopods ingest decayed plant material of different plant types (Wieser 1984). However, experiments of feeding preference between more than two food sources are difficult to analyze (Wieser 1984, Peterson and Renaud 1989).Although the feeding rates of isopods on different plant species have been studied extensively, the reported digestibility efficiency values remain controversial for high and low quality food. Many researchers hypothesize that some foods might present high digestibility values due to a slower passage through the gut, which decreases fecal production (Souza et al. 1998, Loureiro et al. 2006). Changes in the chemical composition of the litter due to decomposition increase its palatability to detritivores (Cameron and LaPoint 1978, Neuhauser and Hartenstein 1978, Rushton and Hassall 1983, Hassall and Rushton 1984, Hassall et al. 1987, Wan Wensen et al. 1993) because the phenolic content decreases during leaf senescence due to the action of microorganisms and leaching (Zimmer 1999, Zimmer 2002a).

Phenolics are thought to play a fundamental role in the chemical defense of plants against herbivores and pathogens (Harborne 1993, Kefeli et al. 2003, Gould and Lister 2006), although their effects are still debated and not fully understood (Appel 1993, Johnson and Felton 2001). The total phenolic content varies with plant growth and abiotic factors, such as temperature and radiation (Salgado et al. 2008), and may affect microbial decomposers since most phenolics remain present during leaf senescence and after death (Bärlocher and Graça 2005). Although poorly understood, the existence of a phenolic cycle in the plant-soil system has been recorded (Kefeli et al. 2003), and differences in the composition and concentration of resin acids and phenolics during leaf and needle litter senescence are known (Kuiters and Sarink 1986, Kainulainen and Holopainen 2002). For example, Hassall and Rushton (1984) observed a negative correlation between isopod feeding preference and the phenol content. In contrast, Neuhauser and Hartenstein (1978) found no relationship between leaf palatability and total phenolic content, and Kasurinen et al. (2007) found a weak or inconsistent correlation between detritivore feeding performance and chemical parameters of leaf litter.

The ability to digest phenolic polymers such as tannins and lignin is essential in the use of litter (Zimmer 2002b), and studies have demonstrated that isopods are capable of oxidizing (Stevenson 1961, Zimmer and Topp 1998, Zimmer 1999b, Zimmer et al. 2002) or hydrolyzing ingested phenolics (Zimmer 1999, Zimmer et al. 2002). For example, Cameron and LaPoint (1978) observed senescence-related decreased mortality and increased leaf consumption after leaching of tannins in *Armadillidium vulgare* (Latreille, 1804), and suggested that litter resources cannot be used immediately after leaf fall due to the chemical and mechanical defenses of plants.

Most studies examining detritivore feeding and phenolics have explored the relationship with total phenolics (concentrations at which animals avoid feeding), lignin content related to toughness (not included in the total phenolic determination since it is a non-soluble phenolic) (Graça and Zimmer 2005), or the capacity of tannins (polyphenolics) to inhibit enzyme catalyzed reactions or to bind and precipitate proteins (Graça and Bärlocher 2005).Conversely, for insects, flavonoids, which are phenolics commonly found in plants, are related to feeding deterrence and might interfere with feeding, molting, and reproduction (Oberdörster et al. 2001, Simmonds 2001, Boué and Raina 2003, Gould and Lister 2006). Some flavonoids play an important role in the protection of plants from harmful ultraviolet (UV)-B levels (Gould and Lister 2006), and several classes of flavonoids show antioxidant activity towards a variety of readily oxidizable compounds (Gryglewski et al. 1987, Dixon and Steele 1999, Zhishen et al. 1999). However, the effects of ingesting flavonoids by other groups of soil invertebrates, such as terrestrial isopods, have received little attention. Studies of isopod nutrition have mainly been conducted in Europe (Rushton and Hassall 1983, Gunnarsson 1987, Szlávecz and Pobozny 1995, Sousa et al. 1998, Zimmer 2002, Ihnen and Zimmer 2008) using species such as *Porcellio* Latreille, 1804 and *Armadillidium* Brandt, 1833, which exhibit worldwide distributions. Studies with neotropical species are relatively uncommon and should be encouraged.

The goal of this study was to observe how two interconnected food parameters (phenolic content and decomposition stage) affect feeding rates (consumption, egestion, and assimilation rates, as well as digestibility efficiency) of detritivores using the neotropical terrestrial isopod *Balloniscus sellowii* (Brandt, 1833) as a model. We relate the total phenolic content to feeding rates and examine the flavonoid contents, which constitute a specific group of phenolics that are known to deter insects. Finally, we test a new method of calculating digestibility (assimilation efficiency) that takes into consideration food retention in the gut.

## Material and methods

### Species and source site

The species *Balloniscus sellowii* (Brandt, 1833)is common in Southern Brazil, Uruguay, and the region surrounding Buenos Aires in Argentina (Schmalfuss 2003). Specimens of *Balloniscus sellowii* were collected in a urban area of Porto Alegre, Rio Grande do Sul, southern Brazil and kept in laboratory conditions at 20 ± 1ºC under a 12:12 (light:dark) photoperiod. Only intermolt animals heavier than 25 mg were used in the experiments, excluding ovigerous females.

The source-site consisted of an area where animals were abundant and there were trees characteristic of pioneer vegetation colonization. The three most abundant plant species in the site were *Lithraea brasiliensis* Marchand (Anacardiaceae), *Ricinus communis* Linnaeus (Euphorbiaceae), and *Schinus terebinthifolius* Raddi (Anacardiaceae).

### Feeding preference

Leaves from the visually most abundant local plants were offered to isopods to verify feeding preference based on the highest consumption. Green leaves from three different plant species were removed from branches and placed into litter bags (10 x 15 cm) fastened to the soil for decomposition in loco for 14 days. Leaves were then transported to the laboratory and circles of 18 mm in diameter were cut and oven dried at 60ºC for 48 hours. The discs were weighed (Gibertini E425-B) and remoistened with distilled water before being offered to animals for one week in individual units consisting of 8 cm diameter plastic containers with moist plaster of Paris covered with a net to minimize coprophagy. The treatments consisted of 10 units with one leaf disc of each plant species with one isopod, and five animal free control units. Animals were kept without food for two days prior to and after the experiment to empty gut contents. After the experiment, the remaining plant material and feces were oven dried and reweighed, and consumption rates were calculated. The control group consisted of units containing leaves and no animals, such that the mean percentage of leaf weight lost due to autogenic changes (weight lost independent of the action of consumers) was subtracted from the amount of plant consumed. The plant species that was consumed at the highest rate was used to verify feeding rates on leaves at different stages of decomposition, as well as the phenolic and flavonoid contents of the leaves.

### Feeding rates on leaves in different stages of decomposition

Green leaves were collected from branches at the same site and placed into 20 litter bags. The litter bags were collected after one, two, and three months of decomposition. These leaves were then taken to laboratory along with additional green leaves that had been collected from the branches when litter bags were placed in the soil, and offered to animals. Oven dried leaves from each decomposition stage were stored under refrigeration for phenolic and flavonoid content analysis.

For each unit, two or three discs of 18 mm (approximated amount for the third month of decomposition, at which point the leaves were very fragmented) were oven dried, weighed, remoistened with distilled water, and offered to individual animals for 10 days. The remaining leaves and feces were collected from the units, oven dried, and weighed after the experiment to calculate feeding rates. We performed 20 repetitions with one animal per unit and 20 control repetitions for each stage of decomposition.

Consumption rates were calculated as the total mg of ingested leaves (subtracted mean percentage of autogenic losses) in dry weight (DW) per g of body weight (FW) per day. The egestion rate was calculated as the total mg of produced feces (DW) per g of body weight (DW), per day. The assimilation rate was calculated as the total mg of ingested leaves (DW) minus the total mg of produced feces (DW) per g of body weight (FW) per day (DW = dry weight; FW = fresh weight) (Loureiro et al. 2006).

### Time of passage through gut and digestibility

We also measured the amount of time that the food was retained in the gut. Animals were kept in individual units containing carrot as a food source (generates fecal pellets that differ in color) for a week. Then, 10 animals were exposed to one disc of leaf litter for each decomposition stage, and monitored every two hours for 80 hours. We recorded the timing of the first sign of leaf consumption (i.e., evidence of nibbling on the leaf disc) and that of the first non-carrot feces appearance.

Digestibility was calculated using animals fed carrots for one week and placed into units containing one leaf disc and monitored daily until total consumption. Following full disc consumption, animals were fed carrots to maintain egestion of the leaf material from the gut. Feces from leaf feedings were collected, oven dried, and reweighted. Five units from each decomposition stage were used. The digestibility was calculated as a percentage based on the total mg of ingested leaf minus the total mg of feces produced per mg of ingested leaf. Given the variability in duration with this method, other feeding rates were not calculated.

### Phenolic and flavonoid content

The total phenolic content was determined using the Folin-Ciocalteau method (Bärlocher and Graça 2005) with tannic acid as standard. For each stage of decomposition, total phenolics were extracted from four samples of approximately 100 mg of ground up dry leaves in 5 mL of acetone for 1 hour at 4°C for a total of 20 samples.

Five samples of each decomposition stage were used to determine the flavonoid content. For each sample, five discs of dry leaves (or an approximate amount) were ground up and left for two days in 5 mL of ethanol 80% for flavonoid extraction. Flavonoid content was determined using the method reported by Zhishen et al. (1999) with modifications using quercetin as standard.

The mean concentrations of phenolics and flavonoids were multiplied by the consumption rates to estimate the total ingested amount of each group of substances.

### Statistical analysis

All data were tested for normality using the Kolmogorov-Smirnov test. The consumption, egestion, and assimilation rates were compared using a one-way analysis of variance (ANOVA) followed by Tukey’s test. Pearson correlations were used to verify the association between consumption and egestion rates among treatments. All statistical analyses were performed using InStat 3.01 software.

## Results

### Feeding preference

**Figure 1.**
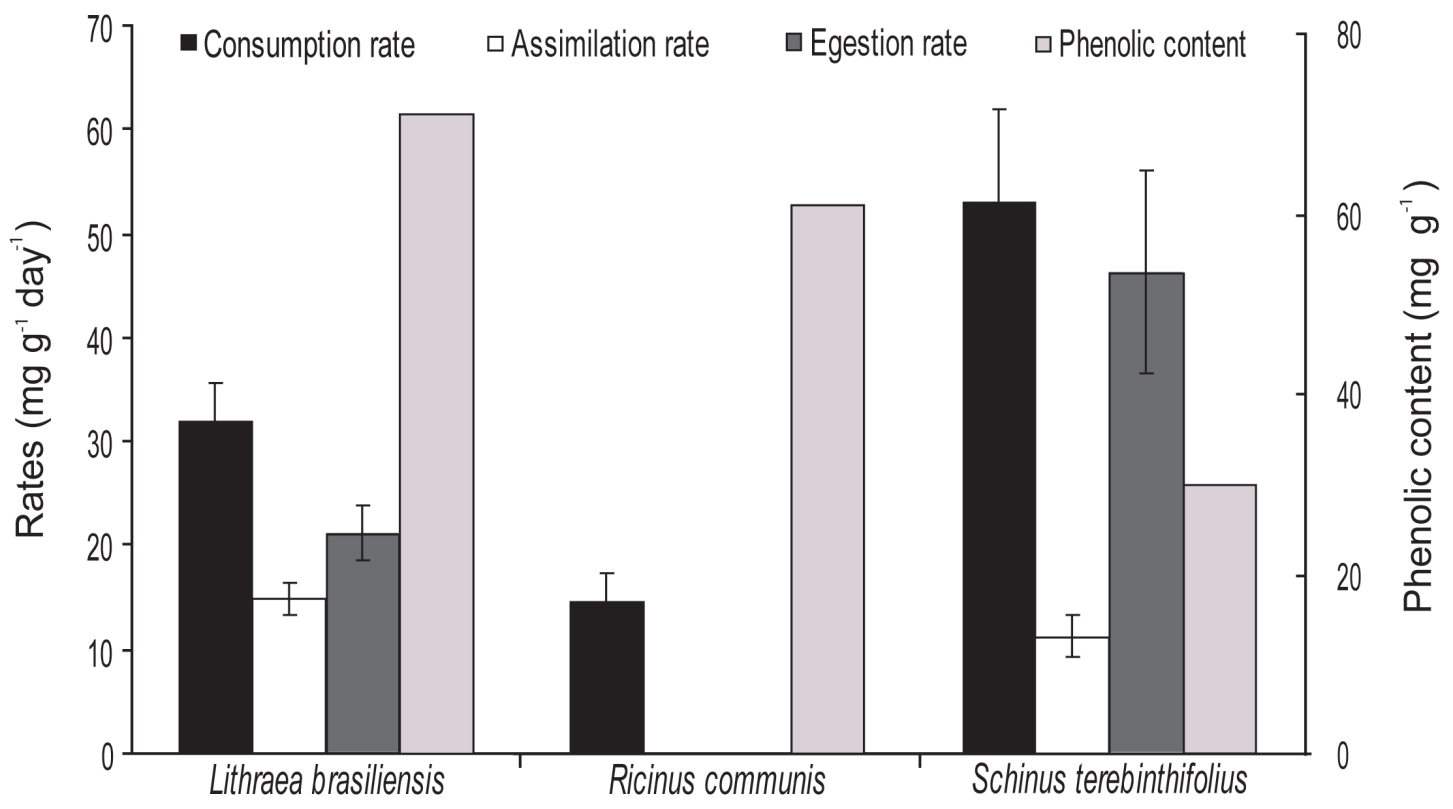
Isopod feeding rates on leaves of *Lithraea brasiliensis* (n = 10), *Ricinus communis* (n = 9), and *Schinus terebinthifolius* (n = 10) with 14 days of decomposition and respective phenolic content (standard error was not calculated due to the low amount of leaf remains for chemical analysis). Egestion and assimilation rate could not be calculated for *Ricinus communis* (low amount of fecal pellets). The values are mean and SE. Superscript letters indicate significant difference among treatments (p < 0.05).

The consumption rate was significantly higher when animals fed on *Schinus terebinthifolius* (52.9 ± 9.0 mg g^-1^ day^-1^,n = 10; mean ± SE) (F_2,26_ = 9.395; *p* < 0.001), and no significant difference was recorded when animals fed on *Lithraea brasiliensis* (31.8 ± 4.0 mg g^-1^ day^-1^, n = 10) and *Ricinus communis* (15.5 ± 2.9 mg g^-1^ day^-1^, n = 9). The egestion and assimilation rates were 46.3 ± 9.8 (mg g^-1^ day^-1^) and 11.2 ± 2.0 (mg g^-1^ day^-1^) for *Schinus terebinthifolius*, and 21.1 ± 2.6 and 14.8 ± 1.62 (mg g^-1^ day^-^1) for *Lithraea brasiliensis*, respectively. Egestion and assimilation rates could not be calculated for *Ricinus communis* due to a low amount of fecal pellets. The phenolic content was highest in *Lithraea brasiliensis* (71.1 mg of tannic acid equivalent per g of dry leaf), followed by *Ricinus communis* (60.9), and was lowest in *Schinus terebinthifolius* (30.0) (Fig. 1). Standard error could not be calculated due to insufficient leaf material for additional replicates. The mass loss of leaves in control units was 0.08% for *Schinus terebinthifolius*, 0.09% for *Lithraea brasiliensis*, and 0.034% for *Ricinus communis*. Mortality was 20% or lower in all treatments.

### Feeding rates on leaves in different stages of decomposition

*Schinus terebinthifolius* was used to examine the feeding rates at different decomposition stages. The consumption rate was significantly higher on two month-old leaves (F_3,58_ = 8.96; *p* < 0.0001), and there were no significant differences between green, one-month-old, and three month-old leaves. The egestion rate was significantly higher for two-month-old leaves (F_3,58_ = 14.17; *p* < 0.0001) and there was no significant difference between green and one-month-old leaves, or between one-month-old and three-month-old leaves. The assimilation rate of green leaves was significantly higher than that of two- and three-month-old leaves and exhibited no significant difference between one-, two-, and three-month-old leaves (F_3,58_ = 5.275; *p* = 0.0028) (Table 1). The mean reduction in leaf mass in the control units was 0.16% (green), 0.13% (one-month-old), 0.12 (two-month-old), and 0.06% (three-month-old). Mortality was 0.35% for green, 0.15% for one-month-old, and 0.20% for two- and three-month-old leaves.

There were significant correlations between consumption and egestion rates for all decomposition stages. The correlation was stronger for one-month-old leaves (r^2^ = 0.928; *p* < 0.0001) followed by those for three-month-old leaves (r^2^ = 0.9258; p < 0.0001), two-month-old leaves (r^2^ = 0.8342; p < 0.0001), and green leaves (r^2^ = 0.7240; p < 0.0002).

**Table 1.** Feeding rates of *Balloniscus sellowii* on *Schinus terebinthifolius* for different stages of decomposition. Data are expressed as mean value and SE of mg of food source (DW), per g of animal (FW), per day. N differs among decomposition stages due to different mortality in treatments. Different letters indicate significant differences of each rate among treatments (p < 0.05).

**Decomposition stage**	**Consumption rate**	**Egestion rate**	**Assimilation rate**
Green leaves (n=13)	41.5 ± 5.1 ^a^	10.7 ± 3.2 ^a^	30.7 ± 2.9 ^a^
1 month-old leaves (n=17)	52.2 ± 4.6 ^a^	29.1 ± 5.7 ^a,b^	23.0 ± 1.7 ^a,b^
2 months-old leaves (n=16)	80.1 ± 6.2 ^b^	61.3 ± 6.4 ^c^	18.9 ± 2.6 ^b^
3 months-old leaves (n=16)	53.4 ± 5.5 ^a^	33.4 ± 5.1 ^b^	20.0 ± 1.5 ^b^

### Time of passage through gut and digestibility

The time required for passage through the gut did not differ among stages of decomposition. For all stages, the mode time for the appearance of leaf-based feces was two hours (one observation interval). However, leaf ingestion was initiated immediately for most units for the one-, two-, and three-month-old leaves, whereas the ingestion of the green leaves was initiated two days after the onset of the experiment. For 3 out of the 10 units containing green leaves, no apparent consumption had occurred after 80 h of observation.

The digestibility, calculated based on the total consumption of a leaf disc with a known mass and a second food source to push leaf material out of the gut, was 43.1 ± 3.8% for green leaves (n = 6), 19.7 ± 2.4% for one-month-old leaves (n = 4), 20.3 ± 1.6 for two-month-old leaves (n = 5) and 19.5 ± 2.8% for three-month-old leaves (n = 2).

### Phenolic and flavonoid content

**Figure 2.**
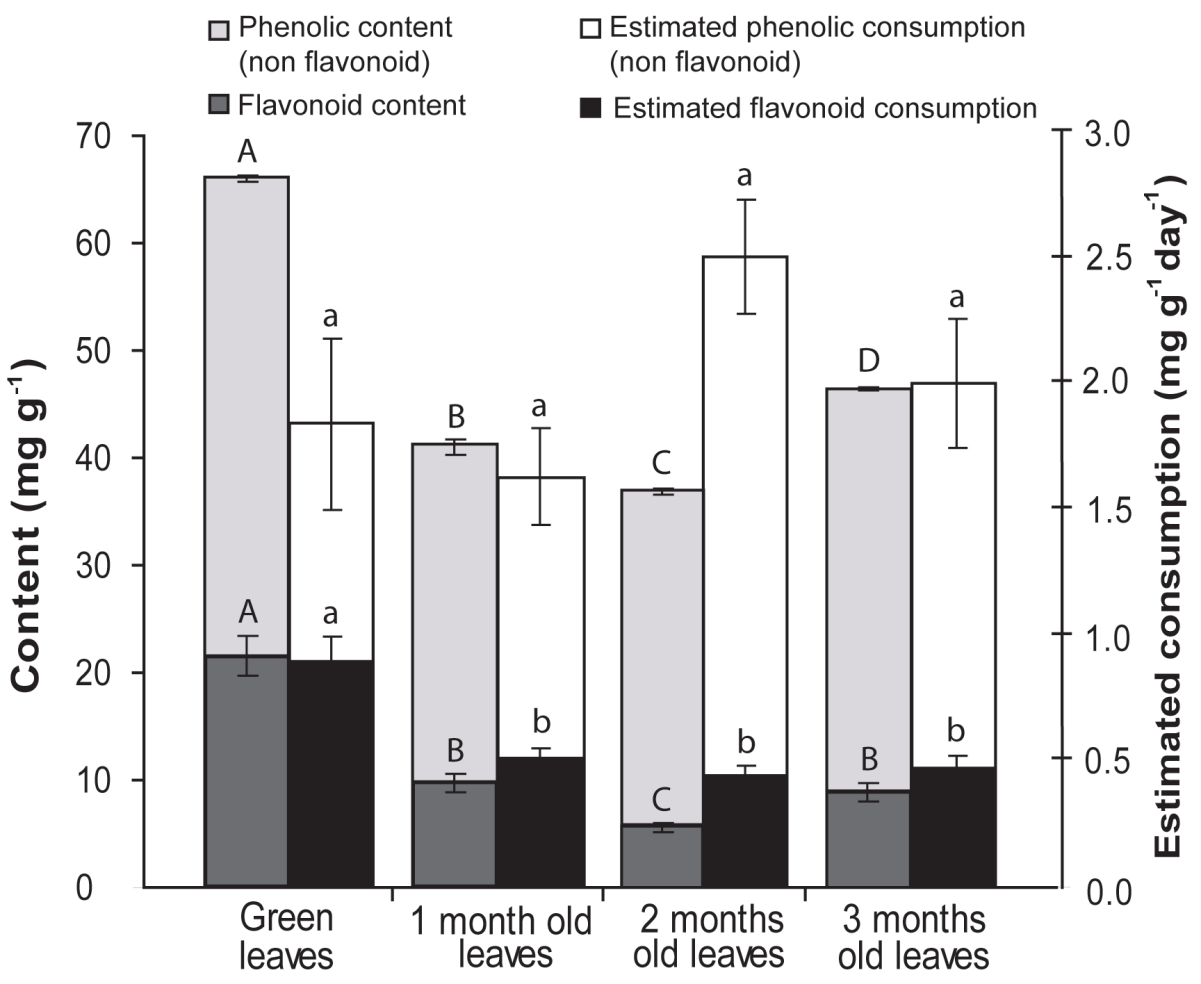
Total phenolic and flavonoid content and estimated amount of total phenolics and flavonoids ingested by *Balloniscus sellowii* on leaves of *Schinus terebinthifolius* for different stages of decomposition. The values are mg of equivalent of quercetin(flavonoid) or tannic acid (phenolic) per mg of dry leaf ± SE. Superscript letters indicatesignificant differences among treatments (p < 0.05).

The phenolic content was significantly different across all decomposition stages (F_3,12_ = 602.61; *p* < 0.0001), and was highest in green leaves (66.0 ± 0.3 mg of tannic acid equivalent per g of dry leaf) and lowest in two-month-old leaves (36.9 ± 0.4 mg g^-1^). The flavonoid content was significantly highest in green leaves (21.6±1.9 mg g^-1^), but did not differ significantly among the other stages (F_3,16_ = 37.10; *p* < 0.0001). The estimated phenolic amount ingested by animals was not significantly different (F_3,58_ = 2.065; *p* = 0.115), ranging from 2.141 ± 0.190 mg of phenolic per g of isopod per day (one-month-old) to 2.954 ± 0.229 (two-month-old). The estimated flavonoid amount ingested by the animals followed the same trend as the assimilation rate among decomposition stages, being significantly higher in green leaves, but not differing between those undergoing one, two, and three months of decomposition (F_3,58_ = 10.783; *p* < 0.0001) (Fig. 2).

Given that the flavonoid content was not tested for every experimental unit, the average content of each leaf age was correlated with the average assimilation rate for each stage of decomposition, resulting in a high correlation (r^2^ = 0.9688; *p* = 0.0157, n = 4).

## Discussion

Numerous studies have analyzed the effects of secondary metabolites in herbivores, whereas few studies have been conducted to understand the role of these compounds in detritivore and decomposer organisms. For example, an understanding of the presence of unpalatable or indigestible compounds and their rates of consumption related to leaf senescence is lacking. Our source site harbored plant species characteristic of a successional stage that do not exhibit mechanical structures to deter herbivores other than lignin, suggesting that chemical defenses are key for plant protection. Tropical plants inhabiting resource-poor environments invest heavily in chemical defenses such as phenolics (Agrawal 2006). *Ricinus communis* exhibited the lowest consumption rate and constituted a small percentage of the fecal pellets egested. This plant has been associated with large amounts of secondary metabolites, including gallic acid, quercetin, and rutin, which represent some of the major phenolic compounds responsible for the antioxidant activity of its dry leaves (Singh et al. 2009). Its decomposition after two weeks resulted in viscous leaves that would be avoided by animals in natural conditions. *Lithraea brasiliensis*, despite being readily found within leaf litter in Brazil, also exhibits a large amount of secondary metabolites (Correia et al. 2006), and its consumption by *Balloniscus sellowii* was significantly lower than that of *Schinus terebinthifolius* and did not differ when compared to *Ricinus communis*.

*Schinus terebinthifolius* accounted for the highest consumption rate and it is known as a source of terpenoids, simple phenolic derivatives, and flavonols. Furthermore, the anti-oxidant activity of the extract derived from its aerial parts has been described in the literature (Velázquez et al. 2003). Leaf extracts contain triterpene acids (Campelo and Marsaioli 1974), and the ethanolic extract of the leaves is a source of simple phenolics, several flavonoids, xanthones, and free steroids (Lima et al. 2006). Once decomposition is initiated, leaves begin to curl and might be used by animals for shelter as well as for feeding.

When given a choice, animals avoid green leaves that provide high amounts of secondary compounds (Cameron and LaPoint 1978). Ingesting decayed leaves is a behavioral strategy used to cope with chemically defended food, as the total amount of ingested defensive chemicals is reduced. Animals may use this strategy to increase their tolerance to chemically defended food (Glendinning 2007). For example, Roy and Bergeron (1990) observed a small rodent cutting leaves from branches then waiting for decomposition to occur before consuming them. We observed that the consumption rate increased throughout decomposition until the second month, when the highest palatability was observed, after which consumption decreased again in the third month of decomposition. Here, feeding on green leaves was a forced laboratory situation used to provide a comparison at an initial stage, and the leaves were oven dried before being offered to animals. This may have changed the leaf properties (as opposed to freshly fallen leaves), thereby allowing feeding. In addition, the decomposed leaves used in this study were handpicked from the trees rather than naturally senescent. However, storm events in this area are frequent, which allow broken branches to undergo decomposition and become available to isopods without undergoing senescence.

In general, detritivores show low digestibility, although differences in the digestibilities of high and low quality foods remain debated. In our feeding rates experiment, green leaves presented very high digestibility values (~80%, data not shown) and there was no difference in the time required for leaf-based feces to appear, contrary to the hypotheses of other researchers (Souza et al. 1998, Loureiro et al. 2006). However, upon calculating the assimilation efficiency based on the total consumption and egestion of a leaf disc and a second food source to push previous food through the gut, the digestibility values for green leaves were lower than those for decomposed leaves. The passage rates of leaves at different decomposition stages did not differ significantly when animals were fed continuously, and leaf-based feces always appeared within the same day that feeding was initiated. However, it is possible that the amount of food retained in the gut under starvation conditions differs with food quality. Therefore, using a second food source to empty the animal gut of previously ingested food makes the estimate of digestibility more accurate. The digestibility values were higher when animals consumed green leaves, which was expected given that digestibility should decrease as decomposition progresses (Rushton and Hassall 1983, Hassall and Rushton 1984) due to the lower content of metabolites in decomposed leaves, which makes them more palatable (Johnson and Feldon 2001). Leaves having undergone one, two, and three months of decomposition exhibited similar digestibility efficiencies and assimilation rates. The assimilation rate is calculated by day and is less affected by the food stored in the gut during starvation at the end of the experiment than the digestibility.

Although the consumption rate did not differ significantly between green and one-month-old leaves, the onset of feeding on green leaves was not immediate, whereas it was for the other stages, suggesting that the high phenolic and flavonoid concentrations of green leaves are feeding deterrents and therefore reduce leaf palatability. These phenolic substances are probably lost in the beginning of the decomposition process due to leaching. If substances that cause feeding deterrence and inhibit feeding are lost early in the leaf decomposition process, the leaves will be consumed more quickly by detritivores, and thus be returned to the soil to provide nutrients back to the trees. Therefore, this process may serve as an adaptive advantage to the plants. Indeed, Cameron and LaPoint (1978) observed rapid leaching of tannins, which was associated with food inhibition from litter bags in the first week of decomposition.

During leaf senescence, the total secondary metabolite content tends to diminish due to leaching (Kuiters and Sarink 1986). Phenolics and flavonoids in plants are largely related to defense against pathogens and herbivores (Dixon and Steele 1999, Boué and Raina 2003), and leaves presenting a lower content of total phenolics are less toxic to isopods. Hassall and Rushton (1984) predicted that less heavily defended species would reach optimal palatability earlier than the climax species, which usually present more secondary metabolites for defense. In our study, the highest consumption was observed in two-month-old leaves, when the total phenolic and flavonoid content was lowest, even though it is thought that the phenolic signature rather than the total phenolic content determines detritivore consumption (Zimmer et al. 2005). The total phenolic and flavonoid content in this experiment increased in the third month rather than decreasing. Leaching of substances also occurs in other materials in the litter, and might increase the content of a specific constituent originating from the litter itself (from absorption) or from the action of microorganisms.

Flavonoids represented a considerable portion of the total phenolics in *Schinus terebinthifolius* leaves. We observed a correlation between the flavonoid content and the assimilation rate, whereas no correlation was detected with the total phenolic content. Although the flavonoid content of the leaves differed among decomposition stages, as did the consumption rates, the estimated amounts of flavonoids consumed by the animals were almost the same for leaves after one, two, and three months of decomposition. Thus, given that a decrease in the consumption of high flavonoid leaves is not supported due to the significantly higher ingestion of flavonoids in green leaves, it appears that the animals increased their consumption of low flavonoid leaves, therefore suggesting that they might use these flavonoids as a food parameter.

Existing reports attribute the presence of phenolics and flavonoids in plants to defense against pathogens and herbivores, while only a few studies suggest possible benefits for organisms ingesting these substances. For example, when examining herbivores, Johnson and Felton (2001) also observed lower consumption and digestibility of plants that were overexpressing phenolics, but no significant reduction for growth and no indications of oxidative stress as a causal factor, suggesting a beneficial antioxidant property for herbivores. This study showed similar results, suggesting that the isopods might also be using flavonoids as an antioxidant agent. Even though the use of flavonoids by herbivorous invertebrates is not well documented, our data suggest that isopods may also use and discriminate concentrations of flavonoids, given that they appeared to increase consumption to minimize their intake of leaves with lower flavonoid contents. It is generally assumed that there is a maximum concentration of feeding deterrents that isopods can tolerate; however, additional studies are needed to examine the minimum intake of substances that can be used as a food parameter.
